# Anesthetic Techniques and Cancer Recurrence after Surgery

**DOI:** 10.1155/2014/328513

**Published:** 2014-02-06

**Authors:** Vincenzo Fodale, Maria G. D'Arrigo, Stefania Triolo, Stefania Mondello, Domenico La Torre

**Affiliations:** Department of Neuroscience, Psychiatric and Anesthesiological Sciences, University of Messina, Policlinico Universitario Pad. E, Piano 1, Via C. Valeria, 98125 Messina, Italy

## Abstract

Many of the most common anesthetics are used in surgical oncology, yet effects on cancer cells are still not known. Anesthesia technique could differentially affect cancer recurrence in oncologic patients undergoing surgery, due to immunosuppression, stimulation of angiogenesis, and dissemination of residual cancer cells. Data support the use of intravenous anesthetics, such as propofol anesthesia, thanks to antitumoral protective effects inhibiting cyclooxygenase 2 and prostaglandins E2 in cancer cells, and stimulation of immunity response; a restriction in the use of volatile anesthetics; restriction in the use of opioids as they suppress humoral and cellular immunity, and their chronic use favors angiogenesis and development of metastases; use of locoregional anesthesia compared with general anesthesia, as locoregional appears to reduce cancer recurrence after surgery. However, these findings must be interpreted cautiously as there is no evidence that simple changes in the practice of anesthesia can have a positive impact on postsurgical survival of cancer patients.

## 1. Introduction

Anesthetics are commonly used, although their effects have not yet been fully elucidated. In particular, many of the most common anesthetics are used in surgical oncology, while their effects on behavior of cancer cells are not yet known. A surgical operation is stressful for the body, and several studies have shown that, after surgery, recurrence of neoplastic disease can occur. Therefore, the majority of data emphasize the importance of the *perioperative period* in surgical cancer management. Surgery causes metabolic, neuroendocrine, inflammatory, and immunological stress and determines upregulation of major malignant molecular pathways involved in tumorigenesis [1]. The results of studies *in vitro* and *in vivo* show that the body's response to surgical stress increases the likelihood of metastatic spread of cancer. Surgery also increases chances of tumor growth and metastasis, due to release of circulating cancer cells during surgical resection of cancer and to the inability of the immune system to neutralize them.

The role of anesthetics and analgesics in postoperative cancer recurrence has also been investigated. Although guidelines for appropriate use of anesthetics in surgical oncology have not yet been codified, these drugs are thought to affect evolution of surgically treated tumors.

Some anesthetics show a mutagenic potential and cause growth of preexisting tumor cells, promoting the two main factors behind carcinogenesis: *transformation* and *immortalization* [2–4]. These agents may induce molecular changes in cancer cells, influence proliferation, angiogenesis, and apoptosis [2, 3, 5], and worsen immunosuppression in cancer patients undergoing surgery [1]. Researchers are therefore working to clarify whether it is possible to improve survival and quality of life of these patients thanks to appropriate choices of anesthetic protocols. The aim of this paper is to contribute to the debate with a “state-of-the-art” review on the possible link between anesthesia and cancer recurrence.

## 2. Materials and Methods

The initial questions that need to be answered, which were used to realize this review, are “*What are the effects of anesthetic drugs on cancer recurrence after surgery?*” “*Can locoregional anesthesia and analgesia, compared with general anesthesia and opioid analgesia, reduce the incidence of postsurgical recurrence of cancer?*”. These questions were used as a starting point for deriving search terms and inclusion criteria for retrieving articles. In particular, the names of different anesthetic protocols along with expressions such as *postsurgical cancer recurrence* have been used. The research was confined to two databases: Cochrane Library and MedLine PubMed.

About one hundred articles, published from 2000 to October 2011, including studies *in vitro*, animal models, and clinical trials, were considered. After a careful screening process, 86 articles were considered eligible and reviewed for quality. The screening process took into account factors such as language, publication data, availability of an abstract and full text, relevance, and study type.

## 3. Results

### 3.1. Links between Perioperative Period and Postsurgical Cancer Recurrence

Despite modern therapeutic progress, metastatic disease remains the leading cause of death in cancer patients. Several theories have been developed to explain the high incidence of postsurgical cancer recurrence during the so-called “*quiescence period,*” in which cancer cells are in a nonreplicative phase, preceding subsequent further growth of the tumor [6–9]. In particular, it was noted that the body's response to surgical stress causes the release of chemical mediators, which determine upregulation of malignant pathways, disruption of tumor homeostasis, and promotion of cancer recurrence [10, 11].

Anesthetic management may positively or negatively affect the predicted deleterious effects of the body's response to surgical stress. According to the hypothesis of *immune surveillance*, the immune system recognizes cancer cells as “*nonself*” and tries to destroy them but is not always able to completely eliminate these cells [12]. From this hypothesis, researchers have developed the theory of *immunoediting*, according to which the immune system selects cancer cells more resistant to clearance, by trying to eliminate them [12]. Some cancer cells can escape immune surveillance by altering antigen presentation and secretion of immunosuppressive agents, proliferate indefinitely, and become a clinically manifested lesion.

Surgery causes immunosuppression through secretion of proinflammatory and anti-inflammatory cytokines and promotes the escape of some tumor cells from immune control [12, 13]. The postoperative period is therefore considered the most vulnerable for development of metastases due to suppression of cell-mediated immunity, first line of defense against tumor cells [8, 14].

A deficit of natural killer cells was observed in large samples of patients with colorectal [15, 16], gastric [17], and lung cancer [18], and with tumors from the head and neck region [19, 20], and is associated with a significantly increased morbidity and mortality. Postoperative immunosuppression is related to the influence exerted by neuroendocrine and inflammatory systems and hypothalamic-pituitary adrenal axis [21].

Psychophysical stress seems to be a predisposing factor for cancer [22]; during the perioperative period, levels of stress markers, epinephrine and norepinephrine, have been found to be very high. These neurotransmitters are believed to be responsible for association between stress and cancer progression, as they are involved in the body's response to surgery [21, 23]. In particular, it has been observed that tumor cells express adrenergic receptors type 1 and type 2, through which epinephrine and norepinephrine activate intracellular cascades, which regulate expression of molecular pathways, determining cancer malignancy and invasiveness.

Ovarian tumor cells acquire invasive potential, or ability for uncontrolled proliferation, as catecholamines activate matrix metalloproteinases, leading to reorganization of extracellular space and promoting angiogenesis. Moreover, catecholamines activate STAT-3, transcription signal activation of several genes in response to stimulation of cells by cytokines and growth factors [24, 25], and increase production of vascular endothelial growth factor (VEGF), implicated in neoangiogenesis.

Cancer is not an anarchic cell replication process. Malignant molecular cascades, which govern processes underlying tumor growth, are regulated by various inflammatory mediators, such as cytokines, chemokines, prostaglandins, and cyclooxygenase.

*In vitro* studies have shown that catecholamines promote migration of breast, ovarian, and colon cancer cell lines, by stimulating their respective receptors and suppressing cell-mediated immunity [26–29]. These factors lead to tumor progression through immunosuppression, resistance to apoptosis, and promotion of angiogenesis [30]. The deficit of natural killer cells in the postoperative period is associated with increased levels of IL-6, IL-8, and PGE-2 and decreased production of IL-2 and TNF-alpha, causing a response of T helper lymphocytes type 1 [6].

Finally, in cancer patients, pain management is extremely important. In animal models, pain causes a deficit of natural killer cells and tumor development, stimulating the hypothalamic-pituitary adrenal axis and the sympathetic nervous system [31–33]. In these animals, good pain control resulted in a significant reduction in susceptibility of primary tumor to produce metastases [34].

### 3.2. Effects of General and Locoregional Anesthesia on Cancer Recurrence

#### 3.2.1. Preclinical Data

Regional anesthesia, unlike general anesthesia, could reduce the incidence of metastatic disease [35, 36].

Inhaled anesthetics, in general, can inhibit proliferation of cancer cells in a time-dependent manner and induce late apoptosis of these cells. However, at the same time, they have a negative effect on cytotoxicity of natural killer cells and NK-like cells, such as altering cytokine release [37].

Kawaraguchi et al. [38] tested the effects of isoflurane exposure on apoptosis of human colon cancer cell lines, trying to clarify the role of caveolin-1 (Cav-1) in cell protection. They observed that brief isoflurane exposure leads to resistance against apoptosis via a Cav-1 dependent mechanism [38].

Recently, Jun et al. [39] explored the effect of isoflurane on proliferation, apoptosis, and invasion of head and neck squamous cell carcinoma cell (HNSCC) lines. Isoflurane seems to increase malignancy of these cells. They supposed that isoflurane might enhance tumor development and promote metastasis in HNSCC patients and suggested that it might be more suitable to choose total intravenous anesthesia [39]. Moreover, several scientific articles support the genotoxic potential of anesthetics and particularly of inhaled anesthetics: this genotoxic potential could have negative repercussions on postsurgical cancer recurrence [40].

Intravenous anesthesia is a widely used technique also in surgical oncology, and, consequently, interest in international scientific literature on the potential effects of these drugs on tumor cells, immune system, and cancer recurrence is quite high. Unlike inhaled anesthetics, propofol does not seem to inhibit growth of cancer cells, but their ability of invasion. Although its effect on NK cells is not well defined yet [37], propofol seems to have protective effects regarding development of metastases, as it stimulates activity of NK cells and inhibits cyclooxygenase type 2 and formation of PGE2 in tumor cells [41]. In rats instead, ketamine and thiopental appear to suppress NK cells and increase the risk of cancer recurrence [5], but these negative effects could be due to the interaction of ketamine with alpha- and beta-adrenoreceptors [42].

Midazolam reduces levels of IL-8, a chemotactic factor, which mediates adhesion and migration of neutrophils, a crucial line of the body's defense, and contributes to immunosuppression in humans [43].

Zheng and colleagues [44] conducted a very interesting study on tumor-bearing rats to demonstrate the effects of different anesthetic protocols on antitumor immune surveillance. Rats were divided into three groups. Group K was treated with ketamine, group P was treated with propofol, and group B was treated with a neuraxial blockade. Rats underwent laparotomy, during which tumor cells MADB106 were injected. After 24 hours, Zheng et al. estimated levels and activity of T lymphocytes CD3, CD4, and CD8, CD4/CD8 ratio, and levels and activity of NK cells CD161a. Three weeks after injection of tumor cells, they examined lung metastases. In groups K and P, levels and activity of T lymphocytes and NK cells were significantly reduced and lung metastases substantially increased [44]. Therefore, many researchers have begun to think that the neuraxial blockade, unlike general anesthesia, could preserve antitumor immune surveillance and reduce the risk of developing metastases after surgery, also in humans.

#### 3.2.2. Clinical Trials

In a retrospective analysis of patients undergoing surgery for breast cancer, Exadaktylos et al. [45] have shown that the association of paravertebral block and general anesthesia was associated with a longer disease-free survival time and lower incidence of cancer recurrence. A more recent study on the effect of combined use of paravertebral block and propofol, in breast cancer patients undergoing surgery, showed a reduction of protumorigenic cytokines, IL-1 and IL-8, and an increase of IL-10, an antitumor cytokine [46].

Another group of researchers verified the effects of locoregional anesthesia on breast cancer cells that detach from the primary tumor, producing dynamic microtubular protrusions, called tubulin microtentacles, formed by vimentin and tubulin-alpha-detyrosinated (Glu-tubulin), which promote the reattachment of these cells. Glu-tubulin and vimentin are cross-linked by motor proteins, called kinesins [47].

Yoon et al. [47], based on the fact that lidocaine and tetracaine inhibit kinesins, have studied the role of these proteins in tubulin microtentacle protrusion formation and noted that lidocaine and tetracaine reduced intracellular motility of kinesins leading to a rapid centripetal collapse of microtentacles, therefore inhibiting cellular aggregation and adhesion.

Locoregional anesthesia-analgesia, total intravenous anesthesia with propofol and synthetic opioids, and the association of these two anesthetic protocols seem to attenuate perioperative factors, favoring the occurrence of minimal residual disease after surgical removal of primary tumor.

Ke and colleagues [48] found that total intravenous anesthesia (TIVA), with propofol and remifentanil, suppressed the inflammatory response to surgical stress to a greater extent than inhaled anesthetic protocol balanced with isoflurane, in patients undergoing open cholecystectomy. They measured levels of proinflammatory cytokines at the end of surgery and anesthesia. Levels of TNF alpha, IL-6, and IL-10 were significantly lower in the group of patients treated with propofol and remifentanil, compared with the group treated with isoflurane [48].

Activation of T helper lymphocytes is a key step in anti-infective and antitumor perioperative immune response. Ren et al. [49] evaluated whether propofol or isoflurane can stimulate the activation and differentiation of these cells, selecting a sample of patients with nonsmall cell lung cancer undergoing surgery for lobectomy. Levels of CD4+, CD28+, IFN gamma, and IL-4 were higher in patients treated with propofol, compared with patients who received isoflurane, leading to the conclusion that propofol stimulates the activation and differentiation of T helper lymphocytes in the perioperative period [49].

Prostatectomy performed in epidural anesthesia seems to be associated with a substantially lower risk of biochemical cancer recurrence.

Biki et al. [50] evaluated prostate cancer recurrence in patients who underwent radical prostatectomy with epidural anesthesia/analgesia or general anesthesia and opioid analgesia, coming to the conclusion that risk of cancer recurrence is 57% lower in patients treated with epidural anesthesia-analgesia. Nevertheless, Tsui et al. [51] have come to different conclusions. They followed a group of patients undergoing prostatectomy for adenocarcinoma, for five years after surgery. Patients were treated with general anesthesia alone, or with general anesthesia and epidural block. No difference existed between the two groups in terms of disease-free survival; therefore, further multicenter randomized clinical trials are necessary to verify the ability of epidural anesthesia to positively influence cancer recurrence after radical prostatectomy [51].

Different types of cancer have been explored. Xing et al. [52] tested the effects of epidural anesthesia-analgesia on cell-mediated immunity and levels of stress hormone in patients undergoing lobectomy for esophageal adenocarcinoma. Group A was treated with general anesthesia and intravenous analgesia postoperatively. Group B was treated with general anesthesia and thoracic epidural anesthesia-analgesia during and after surgery: lower levels of lymphocyte subpopulations were achieved faster in patients of group A (4 hours after surgery), compared with those of group B. In patients of group B, levels of CD4 and CD4/CD8 ratios remained higher than in patients of group A. GH, prolactin, and cortisol increased significantly in all patients [52].

General anesthesia combined with thoracic epidural anesthesia may reduce both the body's reaction to surgical stress and adverse effects of surgery on cell-mediated immunity in patients undergoing lobectomy for esophageal adenocarcinoma. Ismail et al. [53] examined the effects of epidural neuraxial blockade on tumor progression also in patients with cervical cancer, treated with brachytherapy and receiving general anesthesia and epidural block. The results do not indicate a lower risk of cancer recurrence nor increased survival in patients treated with epidural anesthesia [53].

Lin et al. [54], instead, showed that patients with ovarian serous cell adenocarcinoma, treated with locoregional anesthesia/analgesia, have better long-term outcomes than patients treated with general anesthesia and opioid analgesia. They analyzed the clinical history of 143 patients, undergoing surgery for ovarian adenocarcinoma. Median survival rates at 3 and 5 years after surgery were, respectively, 78% and 61% in the group of patients treated with epidural anesthesia-analgesia and 58% and 49% in patients receiving general anesthesia and opioids [54], confirming the initial hypothesis that epidural anesthesia-analgesia, in surgery for ovarian serous cell adenocarcinoma, can reduce mortality of patients in the early years of followup.

In addition, results from a retrospective analysis [55] of a Swedish centre on data from 655 patients, who underwent surgery for rectal cancer and who were treated with epidural anesthesia-analgesia, seem very encouraging. Gupta et al. [55] tested whether the use of epidural blockade reduces long-term mortality in patients undergoing surgery. In the control group, treated with general anesthesia and intravenous analgesia, the risk of cancer recurrence was significantly increased compared to the group treated with epidural anesthesia. Furthermore, postoperative mortality was substantially higher in the control group [55].

In another group of patients, intraoperative use of epidural anesthesia, compared to postoperative use, is associated with a decreased risk of cancer recurrence. De Oliveira et al. [56] selected 182 patients with ovarian serous cell adenocarcinoma, undergoing surgery, receiving epidural anesthesia, or maintaining an epidural catheter intraoperatively and postoperatively. Recurrence was documented in 121 patients and the median time to recurrence was 40 months, in particular, 73 months for the group treated with intraoperative epidural anesthesia, 33 months for those treated with postoperative epidural anesthesia, and 38 months for the group that had not receive epidural anesthesia. Therefore, intraoperative use of epidural anesthesia appears to reduce the risk of postsurgical recurrence of this type of cancer and seems to be associated with an increased disease-free survival time [56].

Other retrospective studies of patients undergoing surgery for prostate and colon cancer have shown favorable results for the use of local anesthetics, administered into the epidural space [50, 57].

Finally, in a large-scale study on human melanoma cells of patients who underwent locoregional anesthesia, instead of general anesthesia, the former was found to be an independent predictor of reduced rates of cancer recurrence [58].

### 3.3. Effects of Opioid and Locoregional Analgesia on Cancer Recurrence

The treatment of pain in cancer patients is a mandatory procedure: it improves quality of life and increases patient's compliance to therapy. Many authors have tried to understand if analgesics, used in the perioperative period, may have an effect on long-term outcome of cancer patients undergoing surgery. Adequate pain management in cancer patients is also critical to prevent immune surveillance deficiency against tumor dissemination, induced by surgical stress.

In particular, it is necessary to clarify the effects of opioids on the immune system and cancer cells and to define if locoregional analgesia may effectively have a protective effect in terms of risk of postsurgical cancer recurrence, compared with opioid analgesia.

#### 3.3.1. Preclinical Data

In rats, there is a clear correlation between postoperative pain and development of metastases [34]. In rodent studies, it has been observed that morphine is proangiogenic and promotes growth of breast cancer [59, 60], and in adenocarcinoma 142 models and in Jurkat cells morphine would seem to promote apoptosis [61]. However, in a mouse model it has been found that repeated administration of morphine leads to a reduction of tissue destruction induced by tumor cells [62].

Fentanyl seems to promote the progression of cancer [63], although, unlike morphine, synthetic opioids do not have immunosuppressive effects and appear to stimulate the activity of NK cells [64].

Mathew et al. [65] examined the new role of Mu opioid receptors in the progression of lung cancer, through laboratory analysis. They identified a mechanism that may provide an explanation for these epidemiological findings, based on regulation by Mu opioid receptor (MOR) of tumorigenicity of Lewis lung carcinoma, found in cells and animal models. Expression of MOR was evaluated using immunoblotting and immunohistochemical analysis in human lung tissue and human lung cancer cell lines. LLC cells were treated with peripheral opioid antagonists, methylnaltrexone, or MOR shRNA, and an increase in MOR expression was noted in samples of patients with nonsmall cell lung cancer (NSCLC) and in several human NSCLC cell lines. Agonists morphine (MOR) and enkephalins increase the growth of LLC cells *in vitro*. However, treatment with methylnaltrexone or silencing MOR expression inhibited invasion and growth of LLC cells in 50%–80% of cases [65]. Injection of LLC cells with MOR silenced led to a reduction of 65% of lung metastases, in mice. In addition, different responses to the injection of LLC cells between MOR knockout mice and wild-type mice were found, as the former do not develop tumors of significant size [65]. In addition, a continuous infusion of peripheral opioid antagonist, MNTX, attenuated growth of LLC primary tumor and reduced lung metastases. Authors have shown a direct effect of opiates on lung cancer progression and suggest a possible therapeutic role for opioid antagonists [65].

Recently, Min et al. [66] have tried to determine whether morphine can attenuate expression of adhesion molecules up-regulated by the supernatant of LPS-stimulated HCT-116 colon cancer cells. They observed that ICAM-1, VCAM-1, and E-selectin expressions were significantly lower when morphine was cotreated with LPS. The conclusion was that morphine affects expression of adhesion molecules primarily by attenuating LPS stimuli on tumor cells [66]. Interaction morphine cancer is likely to be articulated in a very complex mechanism. Furthermore, the effects of neuraxial administration of morphine on the evolution of neoplastic disease have not been widely studied.

#### 3.3.2. Clinical Trials

The possibility that Mu opioid receptor antagonists may influence cancer recurrence is a topic of recent interest. This observation was verified in clinical trials, which showed how preoperative and postoperative administration of morphine can prevent systemic dissemination of cancer cells [62, 67], probably through the stimulation of immune response mediated by T lymphocytes [68]. However, epidemiological studies show that there are differences in postoperative neoplastic recurrence for breast and prostate cancer, according to the anesthetic and analgesic protocol adopted. Even if opioids have represented the main treatment of perioperative cancer pain for many years, it has been established that opioids, in particular morphine, inhibit humoral and cellular immune functions in humans [69, 70].

Human studies suggest that regional analgesia may reduce the risk of cancer recurrence after surgery. Sessler et al. [71] verified if the recurrence of local and metastatic disease after surgical removal of breast cancer is lower in patients treated with paravertebral or high thoracic epidural analgesia, in association with sedation or light anesthesia, compared to patients treated with volatile anesthetics and opioids. They examined breast cancer patients stages 1–3, as part of a multicenter trial, in the third phase. This study was initiated in 2008 and an observation time of five years is planned for these 1100 patients. Outcome will be recurrence of neoplastic disease. If results confirm initial hypothesis, these researchers have shown that small changes in analgesic management of cancer patients can greatly improve prognosis and reduce the risk of postsurgical cancer recurrence [71].

Deegan et al. [72] investigated the effects of different analgesic protocols on breast cancer estrogen receptor-negative cells, treating them with serum of patients undergoing mastectomy using different analgesic techniques. They aimed to demonstrate that locoregional analgesia attenuates the body's response to surgical stress and perioperative immunosuppression. Moreover, they aimed to emphasize how locoregional analgesia can inhibit proliferation, migration, and dissemination of breast cancer cells. Patients were selected to receive propofol and paravertebral analgesia or sevoflurane and opioids. Serum of patients who received propofol and paravertebral analgesia inhibited proliferation, but not migration of cell line MDA-MB-231, ER negative, to a greater extent than serum of patients from the control group [72].

Forget et al. [73] have reviewed 327 patients undergoing mastectomy with axillary lymph node dissection to compare effects on cancer recurrence of ketorolac, sufentanil, ketamine, and clonidine. Their analyses showed a lower rate of tumor recurrence in patients receiving ketorolac before surgery. Other analgesics examined were not associated with a reduced risk of cancer recurrence. Therefore, intraoperative administration of ketorolac seems to reduce risk of breast cancer recurrence as it probably preserves functionality of immunity mediated by NK cells [73]. However, the possibility that some analgesics used in the perioperative period, rather than others, could prevent cancer recurrence has not yet been fully verified.

Curatolo [74] published a review to assess whether the addition of regional analgesia to general anesthesia can have a positive effect on medium and long-term outcome of cancer patients as well as effectively reduce postoperative pain. Research was based on systematic reviews, large epidemiological studies, and multicenter clinical trials. Endpoints considered were perioperative morbidity, cancer recurrence, chronic pain and rehabilitation after surgery, and the risk of neurological damage. Preliminary data suggest that locoregional analgesia could reduce incidence of cancer recurrence, but further clinical trials are needed to clarify the significance of this result. Furthermore, epidural analgesia can significantly improve chronic postoperative pain, and rehabilitation time, with a low risk of neurological damage [74].

Snyder and Greenberg [75] have written a well-articulated review, in which they evaluated effects of volatile, intravenous, and local anesthetics, opioids, and FANS on cancer recurrence. Locoregional analgesia would appear to have a beneficial prognostic effect. Retrospective analyses revealed that paravertebral analgesia improves outcome of breast cancer patients, undergoing mastectomy. In addition, epidural analgesia reduces the risk of tumor recurrence in prostate cancer patients, undergoing prostatectomy. Other perioperative factors, which influence prognosis, include blood transfusions, pain, stress, and hypothermia [75].

Wuethrich et al. [76] examined the influence of different anesthetic/analgesic techniques on outcome of patients with prostate cancer, who underwent radical prostatectomy with open technique. They conducted a retrospective analysis of two groups of patients, treated with general anesthesia and epidural analgesia (January 1994–June 1997; *n* = 103), or with general anesthesia and ketorolac-morphine analgesia (July 1997–December 2000; *n* = 158). General anesthesia in combination with epidural analgesia seemed to improve disease clinical progression-free survival. However, they did not find significant differences in the two groups in terms of disease biochemical recurrence-free survival, cancer-specific survival, and overall survival [76].

Looney et al. [77] tested the hypothesis that propofol combined with paravertebral analgesia could attenuate postoperative changes in plasma levels of angiogenic factors and reduce development of metastases in breast cancer patients undergoing mastectomy, to a greater extent than general anesthesia and opioid analgesia. They considered the following angiogenic factors: vascular endothelial growth factor-C (VEGF-C), transforming growth factor-beta (TGF-beta), placental growth factor (PGF), and fibroblast growth factor (FGF). They drew venous blood from two groups of patients immediately, and 24 h after mastectomy, and then analyzed the serum to detect if there were any changes in concentration of these factors, between pre- and post-operative period. Patients treated with propofol and paravertebral analgesia showed a significant alleviation of pain and a reduction of serum concentrations of VEGF-C and TGF-beta, two hours after administration of analgesics, compared to the other group. Therefore, authors concluded that the use of propofol/paravertebral analgesia can effect blood levels of proangiogenic factors in breast cancer patients [77].

Breast cancer is the most common cancer in women, for whom surgery is now treatment of first choice and the most effective. Many authors have examined the effects of anesthetics and analgesics on the body's response to surgical stress and the immune system. Deegan et al. [78] verified if breast cancer patients undergoing mastectomy, treated with propofol and paravertebral analgesia, exhibited reduced levels of protumorigenic cytokines and matrix metalloproteinases and high concentrations of antitumorigenic cytokines, compared to patients receiving sevoflurane and opioid analgesia. They measured pre- and postoperative serum concentrations of 11 cytokines (IL-1beta; IL-2; IL-4; IL-5; IL-6; IL-8; IL-10; IL-12p70; IL-13 IFN-gamma, and TNF-alpha) and 3 MMPs (MMP-1; MMP-3; MMP-9). Patients treated with propofol and paravertebral block, compared to patients treated with sevoflurane and morphine, showed a reduction of postoperative levels of IL-1beta (less than 26% [−15%  to−52%], compared to preoperative ones (less than 4% [−14%  to  2%], *P* = 0.003); an attenuation of increase in MMP-3 (2% [−39%  to  12%] versus 29% [23%–59%], *P* = 0.011); a significant attenuation of increase in MMP-9 (26% [13%–54%] versus 74% [50%–108%], *P* = 0.02); an important increase of IL-10 (10% [5%–33%] versus −15% [20%  to−2%], *P* = 0.001) [78]. Their results suggest that treatment with propofol and paravertebral block influences a minority of cytokines involved in regulation of antitumor immune response in the perioperative period. Further studies are desirable to determine the significance of these results [78].

The role of perioperative administration of epidural analgesia on cancer recurrence and long-term survival of cancer patients undergoing surgery has aroused curiosity in many researchers.

Retrospective clinical studies show a reduction of cancer recurrence in patients receiving perioperative neuraxial analgesia.

Gottschalk et al. [79], therefore, tried to determine the association between perioperative epidural analgesia and cancer recurrence in colorectal cancer patients undergoing surgery. Authors reviewed the clinical histories of 669 patients, who underwent surgery for colorectal cancer between January 2000 and March 2007. They found no association between the use of epidural analgesia and cancer recurrence, although their results suggested that epidural analgesia is associated with a lower recurrence in older patients but not in younger ones. These results appear to contrast those of retrospective studies on surgery for colon, prostate, and breast cancer; probably, the positive effects of regional analgesia on cancer recurrence may depend on the specific type of cancer [79].

In particular, Myles et al. [80] conducted a followup of a long-term controlled clinical trial, where patients were selected to receive general anesthesia, with or without epidural neuraxial blockade, at least in the first three postoperative days. Twenty-three hospitals in Australia, New Zealand, and Asia were involved, and 503 adult patients participated who were undergoing abdominal surgery for tumor resection. The main outcome measure was neoplastic disease-free survival. Follow-up data were available for 94% of participants. The average time to cancer recurrence or death was 2.8 years in the control group and 2.6 years in the group treated with postoperative epidural analgesia; the results were similar [80].

Animal studies and human reviews suggest that surgical stress, pain, anxiety, and certain anesthetics and analgesics may temporarily suppress the immune system and show that, instead, regional anesthesia/analgesia can minimize immunosuppression through the “sparing” effect of opiates. Nevertheless, considering results of this study, the use of epidural block in abdominal surgery does not appear to be associated with improved cancer disease-free survival.

It is known that persistent postsurgical pain affects from 10% to 50% of women undergoing breast surgery. Recently published studies suggest that preincisional paravertebral block can reduce incidence and severity of postsurgical breast pain. However, in practice, no difference between patients treated with paravertebral block and patients receiving general anesthesia was registered.

Opioid requirement was statistically reduced in the group treated with paravertebral block [65].

Finally, Forget et al. [81] conducted a retrospective analysis and analyzed 1111 prostate cancer patients. They observed that intraoperative administration of sufentanil is associated with an increased risk of cancer relapse after retropubic radical prostatectomy, whereas epidural analgesia, with local anesthetics and opioids, was not associated with a significant effect [81].

## 4. Discussion

The aim of this review was to clarify the effects of anesthetics on tumor cells and the immune system and to stimulate the elaboration of international guidelines for selecting the most appropriate anesthetic techniques to improve long-term outcomes of cancer patients.

Although the role of anesthetics in cancer progression has not yet been fully elucidated, in the literature reviewed there is evidence to support that anesthetics affect cancer, immune system, and molecular processes involved in cancer development.

Volatile anesthetics may damage immune functions of neutrophils, macrophages, dendritic cells, T lymphocyte cells, and NK cells. In a recent study on direct and indirect effects of anesthetic agents, Tavare et al. [82] tried to identify pathophysiological mechanisms to explain the influence of anesthetic techniques on postoperative metastatic spread. They demonstrated that some anesthetic agents determine an unregulation of hypoxia inducible factor-1-alpha (HIF-1-alpha) in cancer cells, linked with more aggressive phenotypes and worse outcome [82].

Another group of researchers [83] wished to verify if the duration of anesthesia, in particular sevoflurane anesthesia, can be associated with an increased risk for new malignant disease within 5 years after surgery. No associations were found with duration of sevoflurane anesthesia [83].

Scientific studies support the use of intravenous anesthetics, such as propofol, with the restriction of use of volatile anesthetics; the addition of regional anesthesia might decrease recurrence after cancer surgery [1].

Despite their analgesic power being well established, opioids appear to have several negative effects, not all clearly elucidated, in particular on immune response. There are no reliable data demonstrating that opioids are directly involved in tumorigenesis in humans. However, results of animal studies suggest that these drugs may contribute to cancer recurrence in a clinical setting. The definition of opioid effects on cancer recurrence could help determine the best analgesic approach, curbing the possibility of postsurgical metastatic spread of residual tumor cells. Morphine appears to be proangiogenic and promotes tumor expansion. Despite this, laboratory data on the effects of morphine on cancer recurrence are contradictory and even the few randomized clinical trials do not show any improvement in survival and reduction of cancer recurrence in patients treated with locoregional anesthesia-analgesia rather than with general anesthesia and opioids. Some studies show that morphine has a direct proapoptotic and antiproliferative effect on different tumor cell lines. Furthermore, studies *in vitro* have shown an angiostatic effect for morphine.

Finally, locoregional anesthesia and analgesia seem to reduce incidence of postsurgical cancer recurrence in patients suffering from certain types of tumor.

Locoregional anesthesia prevents the body's neuroendocrine response to surgical stress by blocking transmission of neuronal signals to the central nervous system, as well as those of efferent sympathetic nervous system activation.

Thus, NK cell function seems to be better preserved by locoregional anesthesia; in addition, the possibility of metastatic disease is significantly limited.

Moreover, the combined use of locoregional anesthesia with general anesthesia reduces the amount of general anesthetic required, as well as degree of immunosuppression.

Locoregional analgesia provides better pain control, eliminating the need for opioids in the postoperative period and resulting in negative effects on immune function and tumor growth; it also reduces the release of endogenous opioids.

Results of animal studies support these theories, as they show how regional anesthesia and postoperative local analgesia, regardless, attenuate development of metastasis in animals, inoculated with breast adenocarcinoma cells.

Data from human studies, although still very limited, confirm this hypothesis.

Indeed, paravertebral anesthesia/analgesia, used for surgical resection of breast cancer, is associated with a lower risk of cancer recurrence.

Epidural analgesia, used for radical prostatectomy to remove adenocarcinoma, is correlated with a 60% risk reduction of recurrent neoplastic disease.

Multicenter clinical trials on large samples are needed to confirm existing scientific evidence in this field. Some clinical trials are currently underway to confirm positive effects of paravertebral analgesia on outcome of breast cancer patients, and epidural analgesia on those of colon cancer patients [84–86]. Finally, it is undoubtedly important to verify if local anesthetics can decrease metastatic progression in humans.

In conclusion however, these findings must be interpreted cautiously as there is no evidence that simple changes in the practice of anesthesia can have a positive impact on postsurgical survival of cancer patients.

Further multicenter randomized clinical trials are needed to confirm the hypothesis that postsurgical cancer recurrence can be significantly decreased in patients treated with locoregional anesthesia and not in those receiving only general anesthesia. It is also important to verify whether the association between general anesthesia and locoregional anesthesia may improve the outcomes of cancer patients [71].
